# Cancellation case analysis of physician-staffed rapid response car activities during off-hours in the central region of Saitama prefecture, Japan: a retrospective study

**DOI:** 10.1186/s12873-026-01698-2

**Published:** 2026-07-21

**Authors:** Kaoru Ogawa, Ishtiaq Ahmad, Yoshihisa Shirayama, Motohiro Tsuboi, Koichi Yasaka, Shigemasa Taguchi, Miyoko Okamoto, Kazuya Kiyota, Motoyuki Yuasa

**Affiliations:** 1 Department of Global Health Research, Graduate School of Medicine, Juntendo University, Hongo 2-1-1, Bunkyo City, Tokyo, 113-8421 Japan; 2 Altitude Emergency and Critical Care Medical, Saitama Red Cross Hospital, Saitama, Japan

**Keywords:** Anaphylactic shock, Dispatch cancellation, Pre-hospital emergency care, Rapid response car, Respiratory failure, Saitama prefecture

## Abstract

**Background:**

Among hospitals in the central Saitama Prefecture, only the Saitama Red Cross Hospital (SRCH) provides physician-staffed rapid response car (RRC) services during off-hours (weekday night-time, weekends, and public holidays). During off-hours, concentrated dispatch requests increase the operational and financial burden, particularly when cancellations occur. Furthermore, research on RRC cancellation in the Japanese urban emergency medical system remains limited. By examining the epidemiological characteristics of RRC cancellation, this study aimed to optimise RRC operations and enhance emergency medical response efficiency.

**Methods:**

This retrospective study analysed RRC dispatches from SRCH between January 1, 2021, and December 31, 2023, focusing on cases classified as ‘cancelled’ or ‘treated’. Variables including age, disease type, distance from the hospital, request source, and time of request were compared using chi-square test, *t*-test, and Mann–Whitney U test. Logistic regression was performed to identify independent predictors of cancellation.

**Results:**

Of 4,005 dispatches, 1,091 were cancelled, and 2,417 were treated; 497 cases were excluded because of incomplete data. Cancellations were significantly associated with patients aged ≥ 65 years, requests initiated by the Command Centre, and greater distance from the hospital. Respiratory failure and anaphylactic shock had the highest cancellation rates.

**Conclusions:**

Improving dispatch strategies can enhance pre-hospital emergency care, ensuring timely medical attention for critical patients, while reducing operational and financial strain on medical institutions.

## Background

Japan has a well-developed emergency medical service (EMS) system, with more than 7,000,000 ambulance dispatches annually nationwide, according to the Fire and Disaster Management Agency. In Saitama City alone, more than 86,000 ambulance dispatches occur each year, reflecting the increasing demand for EMS in urban areas. However, detailed pre-hospital data, particularly regarding physician-staffed ground units, are limited. A national registry for physician-staffed rapid-response cars was launched only recently, in January 2024, and comprehensive data collection is still in progress. Consequently, regional analyses, such as the present study, provide valuable insights into the operational characteristics of Physician-staffed EMSs (P-EMSs) in Japan, especially in urban settings where multiple dispatch pathways coexist.

P-EMSs that use helicopters or rapid response cars (RRCs) are widely recognised in many developed countries, particularly in Europe [1–3]. The operational concept of P-EMSs varies across regions because of differences in geographical features, healthcare organisations, and emergency response systems. However, a typical feature is the presence of a pre-hospital care-trained physician who strives to stabilise critically ill or injured patients and transfers them to an appropriate hospital or facility under optimal conditions. Helicopter-based P-EMSs are generally considered more effective in remote areas [4, 5]. In Japan, 57 doctor helicopter units operate under a national commitment to provide high-quality emergency care to all citizens, regardless of their financial situation or geographical location. These units have recorded over 30,000 dispatches annually, primarily for interfacility transport of critically ill patients and long-distance emergency responses [6]. However, despite their ability to travel farther in less time, helicopters are significantly affected by weather conditions and airspace restrictions [2]. In contrast, RRC-based P-EMSs offer greater reliability under varying conditions, particularly in urban settings [7].

Extensive research has been conducted on the impact of P-EMSs on patient outcomes [8–11]. A recent nationwide study comparing physician-staffed air and ground transport in Japan revealed that, despite longer pre-hospital times and fewer in-transit interventions, air ambulance transport was associated with significantly lower in-hospital mortality among severely injured patients. These findings indicate that strategic physician involvement and hospital destination selection may outweigh time savings when optimising patient outcomes [12]. In Japan, there are two primary types of vehicle-based P-EMSs: RRCs and ambulances with physician staffing. RRCs primarily transport medical personnel (doctors and nurses) to emergency situations but lack patient transfer capabilities. Notably, ambulances with physician staffing are equipped to transport patients and may contain advanced medical devices, such as extracorporeal circulation systems [9].

Regardless of the transport method, P-EMS dispatch decisions often rely on incomplete information from emergency calls, often resulting in cancellations after dispatch and wasting of human and financial resources. However, few studies have specifically addressed the cancellation rates of P-EMS dispatches [13–16].

Further research is needed to establish efficient operational strategies that minimise unnecessary cancellations and alleviate the burden on RRC personnel and management institutions. Although multiple hospitals operate RRC services during daytime hours on weekdays in the central region of Saitama Prefecture, Saitama Red Cross Hospital (SRCH) is the sole provider in off-hours. In this study, the RRC operated by the SRCH was a physician-staffed vehicle designed solely to transport medical personnel to the scene and was not equipped to transport patients. All patients requiring transportation were transported by standard EMS ambulances. Notably, Saitama has the lowest number of physicians per 100,000 population nationwide, 180.2 as of 2022, underscoring the disproportionate burden placed on limited emergency medical resources during off-hours. This concentration of responsibility may lead to significant operational and financial burdens, particularly when dispatch requests are unnecessary or cancelled after initiation.

Therefore, this study aimed to investigate the characteristics of RRC cancellations during off-hours in the central region of Saitama Prefecture by analysing patient age, type of illness, distance from the hospital, timing of the request, and request source, with the goal of identifying factors that may improve emergency medical response efficiency and support sustainable RRC operations.

## Methods

### Data sources and RRC system

SRCH is a tertiary care facility with 638 inpatient beds, including 8 beds in the intensive care unit and 14 beds in the cardiac care unit. In 2023, the emergency unit accepted 6,139 secondary emergency care patients and 2,213 tertiary emergency care patients.

**Fig. 1:**
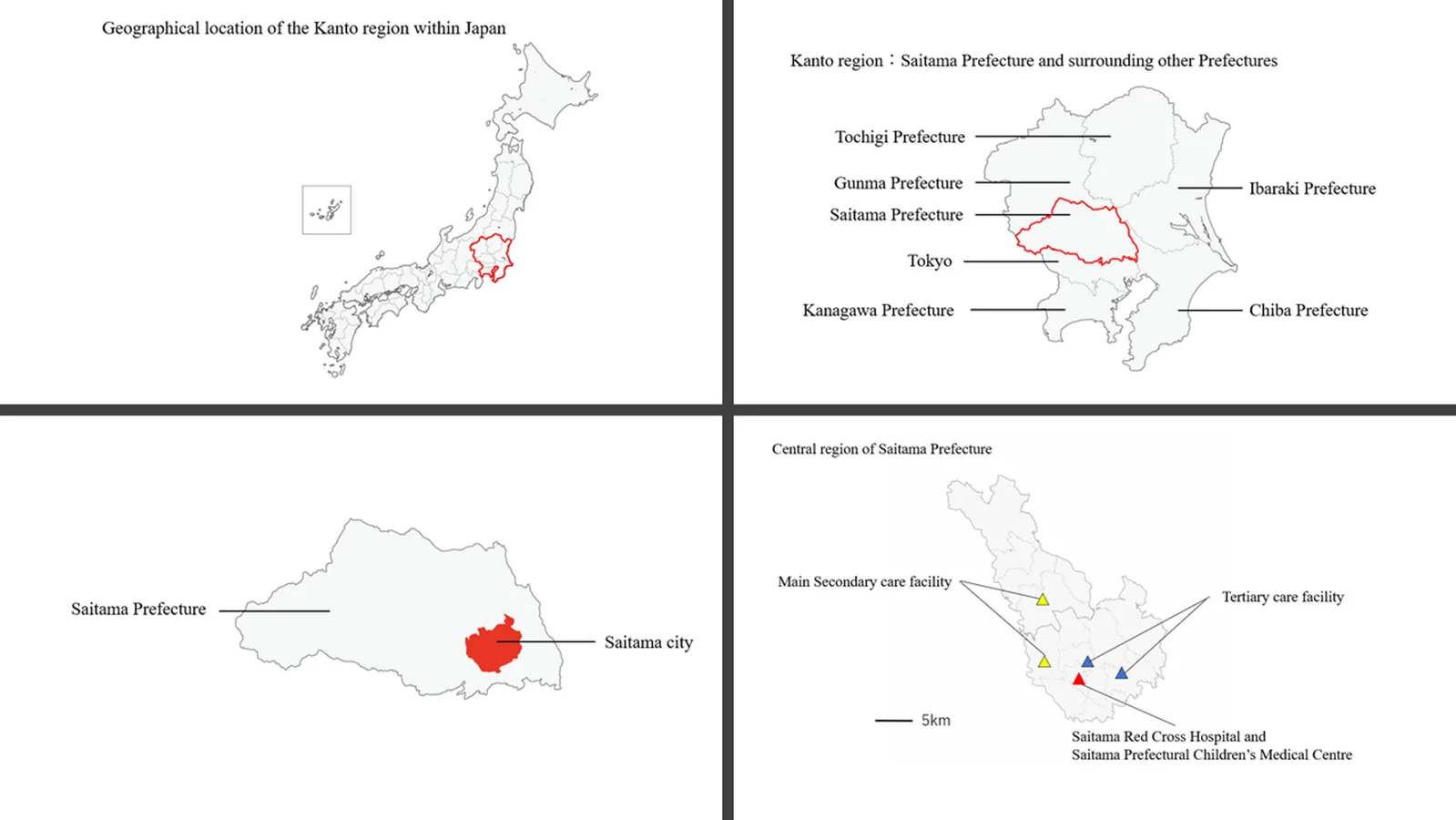
Location of Saitama City and its major medical hospital in Central region of Saitama Prefecture

The SRCH has been operating an RRC covering all districts of Saitama City, Ageo City, Okegawa City, Kitamoto, Kounosu City, Ina Town, and some other nearby cities in Saitama Prefecture. The SRCH is also the only designed trauma and perinatal medical centre in the region. This medical control area comprises a total population of 1,986,726, covering an area of 390.34 km^2^ as of December 2024. A map of the key features of the medical control area is shown in Fig. 1.

Secondary care facilities: Provide inpatient care for moderate‑severity emergency patients who require hospital admission.

Tertiary care facilities: Capable of managing life-threatening conditions and providing advanced interventions such as intensive care, emergency surgery, and multidisciplinary critical care.

These classifications are defined by the Ministry of Health, Labour and Welfare.

**Fig. 2:**
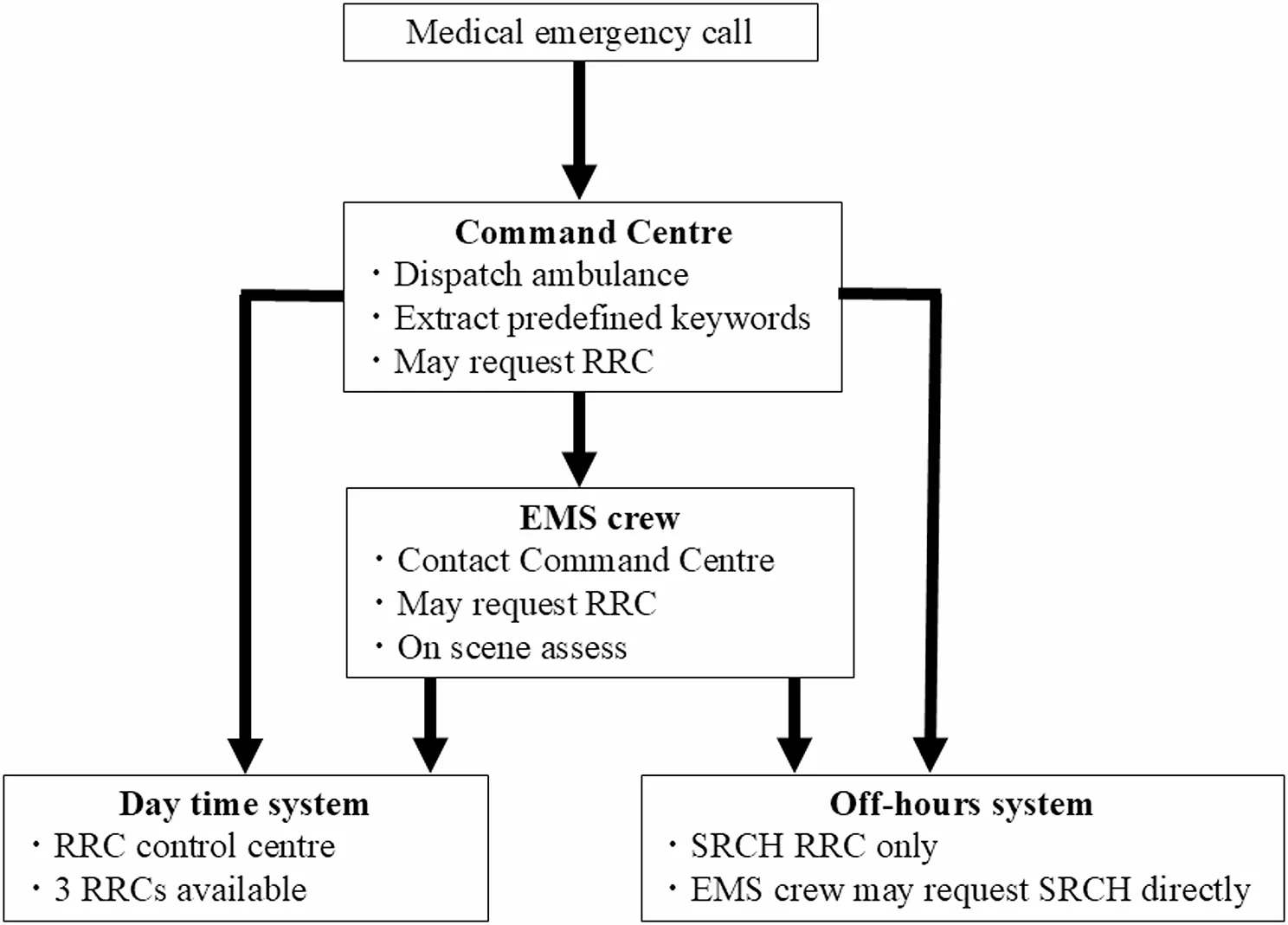
The operating system of the RRC during weekday daytime and off-hours in the central region of Saitama Prefecture

The Saitama City area has four tertiary medical facilities. Among them, the SRCH and the Saitama Prefectural Children’s Medical Centre, which is also a tertiary care facility specializing in paediatric emergency and critical care, are adjacent and jointly operate a physician staffed RRC. This collaboration enables 24-hour, year-round RRC coverage for both adult and paediatric emergencies, while other two tertiary hospitals operate their own RRC units. This system results in three hospitals providing RRC services in the region (Fig. 1). In this region, the organisation of RRC services differs between daytime and off-hour periods. During weekday daytime hours (8:30 to 17:15), RRC requests are coordinated through a dedicated RRC control centre. All three RRCs are in operation. When the Fire Department Command Centre receives an emergency call containing predefined keywords that match the dispatch criteria (Table 1), it contacts the RRC control centre, which determines the most appropriate RRC for dispatch based on factors such as geographical proximity and estimated response time. During off-hours, the RRC control centre is closed, and the SRCH becomes the sole provider of RRC services (Fig. 2).

**Table 1:** Dispatch criteria based on pathological condition

Category	Pathological condition	Keywords
Trauma	- gunshot wounds - stab wounds - explosion accidents - multiple injuries - high-risk injury mechanisms - drowning	- shot - stabbed - explosion accident - multiple injuries - high-risk injury mechanism - drowning - >20 min rescue from vehicle/machine
Cardiac Arrest	- defibrillation performed - witnessed sudden unconsciousness and respiratory arrest - sound of falling, followed by loss of consciousness and respiratory arrest	- defibrillation performed - sudden unconsciousness and arrest - found collapsed
Circulatory Failure	- chest pain > 30 min - breathlessness - impaired consciousness - nausea, vomiting, and cold sweats - severe back pain with a history of hypertension, syphilis, and Marfan syndrome	- continuous chest pain - cold sweat, pale face - older than 40 - severe unfamiliar back pain - profuse sweating
Respiratory Failure	- wheezing, coughing - dyspnoea - severe hyperventilation - cannot lie down or walk - sudden onset dyspnoea with cyanosis or altered consciousness	- difficulty breathing or speaking - asthma history - use of accessory muscles - happened during eating - unable to communicate - clutching the throat/chest
Anaphylactic Shock	- dyspnoea, throat tightness - sneezing, coughing - abnormal breath sounds - red/swollen face - chest discomfort, hypotension - gastrointestinal symptoms (nausea, abdominal pain) - dizziness, lip/tongue numbness - rash, oral swelling/itching	- difficulty breathing or speaking - allergy history - facial swelling and redness
Mass Casualty Incident	- events with ≥ 5 injured individuals	- ≥5 injured persons

Ambulance dispatch is mandatory once a 119 call is received, because ambulances are legally required to respond to all requests under the national EMS framework. In contrast, RRC dispatch requires an additional decision by the Fire Department Command Centre. The initial RRC dispatch decisions are based on predefined keywords extracted from the emergency call; however, these keywords do not cover all clinical presentations. Therefore, ambulance crews may request an RRC on scene when they judge that physician involvement is necessary, even if the initial call does not contain any predefined keywords. Physicians may also initiate an RRC dispatch based on the information provided by the ambulance crew. Once the RRC team and the ambulance crew meet at the scene and begin joint management, the destination hospital is selected collaboratively by the RRC physician and EMS personnel based on the patient’s condition, and hospital availability. Conversely, RRC dispatches may be cancelled after ambulance assessment if the patient’s condition is mild or if transport to another hospital has already been arranged.

This figure illustrates the differences between the daytime and off-hour RRC activation pathways.

### Daytime system (left side)

During weekday daytime hours (8:30–17:15), the Fire Department Command Centre extracts predefined keywords from emergency calls and contacts the regional RRC control centre. The control centre evaluates dispatch eligibility and coordinates RRC deployment among multiple tertiary hospitals operating RRC units. In addition to keyword-based activations, ambulance crews may request an RRC after on-scene assessment or while engaged in an ambulance mission. These requests are relayed either to the Command Centre or directly to the RRC control centre, depending on the situation.

### Off-hours system (right side)

During off-hours, the regional RRC control centre is closed, and SRCH functions as the sole provider of RRC services. The Fire Department Command Centre and ambulance crews contact SRCH directly to determine dispatch eligibility and request RRC deployment.

The RRC crew members at the SRCH typically consist of one or two emergency physicians, a nurse and driver, and sometimes a paramedic. Detailed information, such as the date, age, and sex of the patient; reason for RRC request; location of the scene; occurrence of cancellation; reason for cancellation; and the hospital to which the patient was transported, is recorded after each RRC activity. Notably, RRC requests were documented based on the initial emergency call and not the final diagnosis.

### Study design and selection of participants

A retrospective observational study was conducted at SRCH, reviewing all dispatch requests from the RRC centre and focusing on off-hour RRC activations between January 1, 2021, and December 31, 2023. Patients were excluded if they did not meet dispatch criteria or if essential data, such as sex or age, were missing. Because the study dataset included only RRC dispatches for which SRCH provided the service, weekday daytime dispatches (08:30–17:15) were outside the scope of the present analysis. Daytime RRC coverage is shared among multiple tertiary hospitals, and complete dispatch data from these institutions were not available, precluding inclusion of daytime cases. Including only partial daytime data would have introduced institutional heterogeneity; therefore, to ensure consistency, the present study analysed only SRCH-based dispatches and excluded weekday daytime dispatches. Included cases were categorized into the following two groups based on the occurrence of cancellation: the ‘cancelled’ group, for which dispatches were cancelled after being issued, or the ‘treated’ group (control group), for which physicians on the scene treated patients. The major reasons for cancellation were “Mild condition” and “Starting transport to another hospital”. “Mild condition” referred to cases in which patients had stable vital signs and were considered suitable for transport by EMS personnel without physician accompaniment. “Starting transport to another hospital” indicated situations where the destination hospital had already been determined before the RRC arrived, and EMS personnel judged that prioritising immediate transport rather than waiting for the RRC would be more beneficial. “Others” included cases that did not fall into these categories, such as dispatch errors or changes in the situation reported by bystanders.

### Data collection

We collected the following data by reviewing the dispatch requests: age, sex, and address of the requester, RRC dispatch date and time, activity outcome, delivery destination, reason for request, reason for cancellation, and return time. These were considered independent factors in the analysis. RRC requests originate from command centres, on-site emergency teams, or physicians. Distance refers to the distance between the requested address and the SRCH was calculated by measuring straight-line distances using Geospatial Information Authority maps. For cases with missing return times, the estimated values were supplemented using publicly available mapping tools, and when available, hospital arrival timestamps were incorporated to improve the accuracy. Diseases were categorised into trauma, cardiac arrest, circulatory failure, respiratory failure, anaphylactic shock, mass casualty incidents, and others for analysis (Table 1). The “Others” category included severe conditions not explicitly covered by predefined dispatch keywords, such as acute paediatric illness, obstetric emergencies, burns, gastrointestinal haemorrhage, and endogenous shock states. These cases were classified based on the initial information provided by the emergency call or on-scene assessment when RRC activation was requested, despite the absence of a corresponding keyword.

Regarding the calculation of costs and working time, the crew members of the SRCH were salaried employees, resulting in no additional labour costs for cancelled cases. The mean working time for cancelled cases (referred to as ‘mission time’ in Table 2) was calculated as the average time from dispatch to return arrival at SRCH.

### Statistical analysis

The analysis primarily focused on cancellation cases during off-hours, based on age, disease, distance, time, and request type. Weekday daytime requests were not included in the analytical dataset, because complete daytime data from other RRC-operating hospitals were unavailable. Therefore, all statistical analysis were conducted using off-hour RRC requests from SRCH only, defined as weekday nighttime and holiday daytime and nighttime. National public holidays were defined based on the Japanese Cabinet Office calendar. During the study period, 14 holidays in 2021, 15 holidays in 2022, and 12 holidays in 2023 did not overlap with weekends and were included in the analysis. Continuous variables were presented using both medians with interquartile ranges (IQR) due to their nonparametric distribution and means with corresponding standard deviations (SD) for descriptive purposes. Categorical variables were displayed as raw values, with percentages in parentheses. The chi-square (χ^2^) test, *t*-test, and Mann–Whitney *U* test were used to compare medians between the two groups. The χ^2^ test was specifically used to compare frequencies. The odds ratios (ORs) and 95% confidence intervals (CIs) for cancellation were determined using multivariate logistic regression adjusted for the following covariates: age, sex, distance from the hospital, and requester for the RRC. A two-sided significance level of 5% (*p* < 0.05) was used for all the statistical tests. All analyses were conducted using the STATA software, version 18 (Stata Corp., Texas, USA). Missing values, primarily related to age and sex, were handled using list-wise deletion.

### Disclosure

The study protocol was approved by the Ethics Committee of SRCH (Approval No. 24-C). This study did not involve any invasive procedures or interventions, and only information from medical records was utilised. Individual consent was not obtained from any patient. However, information about the study was prepared, and patients were provided with the opportunity to refuse participation. This corresponds to an opt-out procedure published on the SRCH website under the disclosure of information on clinical research.

## Results

During the study period, 5,652 dispatch requests were recorded, with 4,005 occurring on off-hours, accounting for 70.9% of all dispatch requests. This indicates a significant concentration of RRC activities during non-business hours, highlighting the increased burden on emergency medical personnel during this period.

**Fig. 3:**
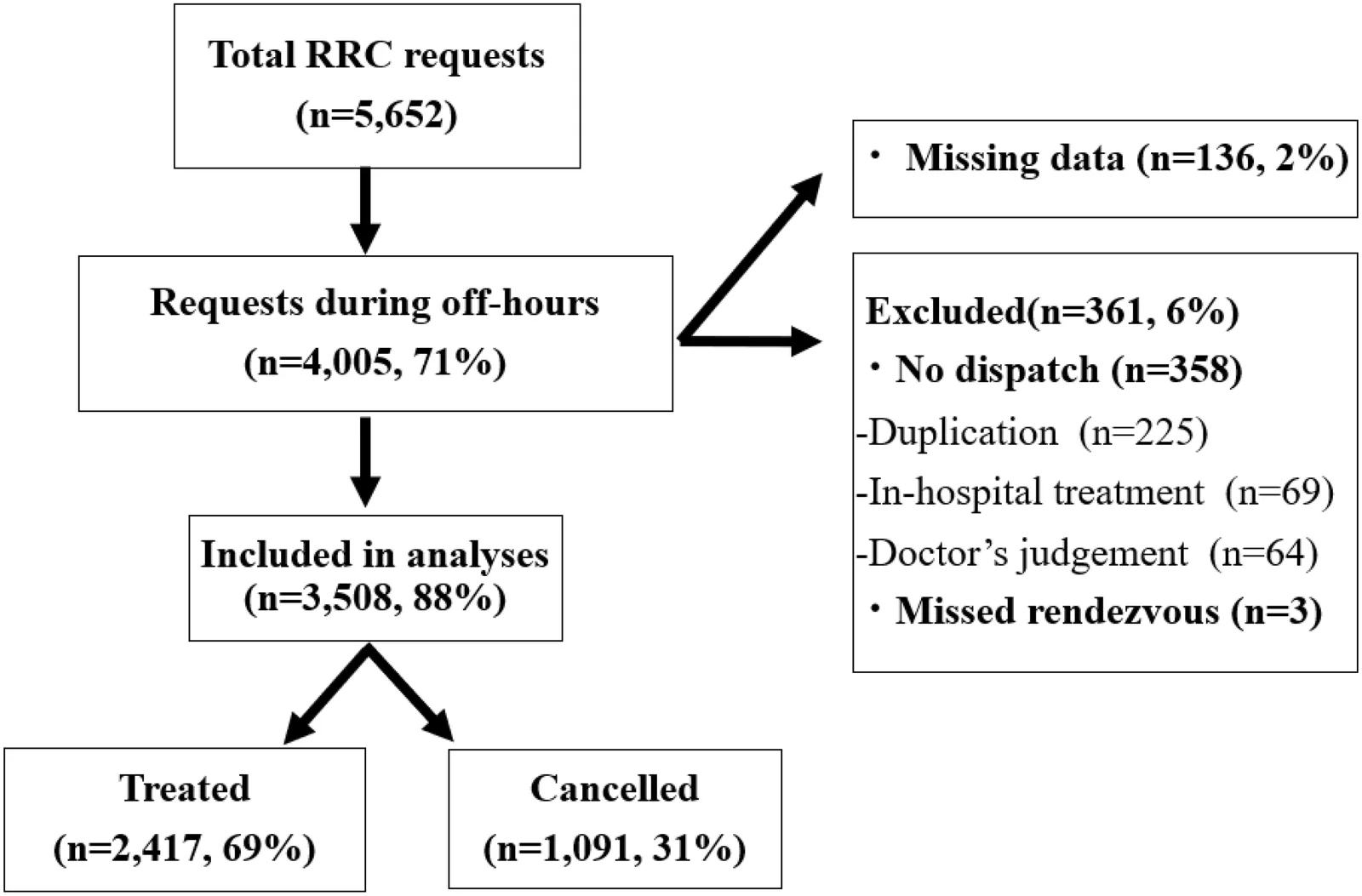
Participant selection flow diagram for this study. Cases were excluded because no dispatch involved duplication (*n* = 225), in-hospital treatment of critically ill patients (*n* = 69), judgement of the doctor (*n* = 64), and missed rendezvous (*n* = 3)

After excluding 497 cases owing to incomplete data or failure to meet the dispatch criteria, 3,508 cases were analysed. Among these cases, 1,091 (31.1%) were classified as the ‘cancelled group’, and 2,417 (68.9%) were classified as the ‘treated group’ (Fig. 3). The primary characteristics of the study participants are listed in Table 2.

**Fig. 4:**
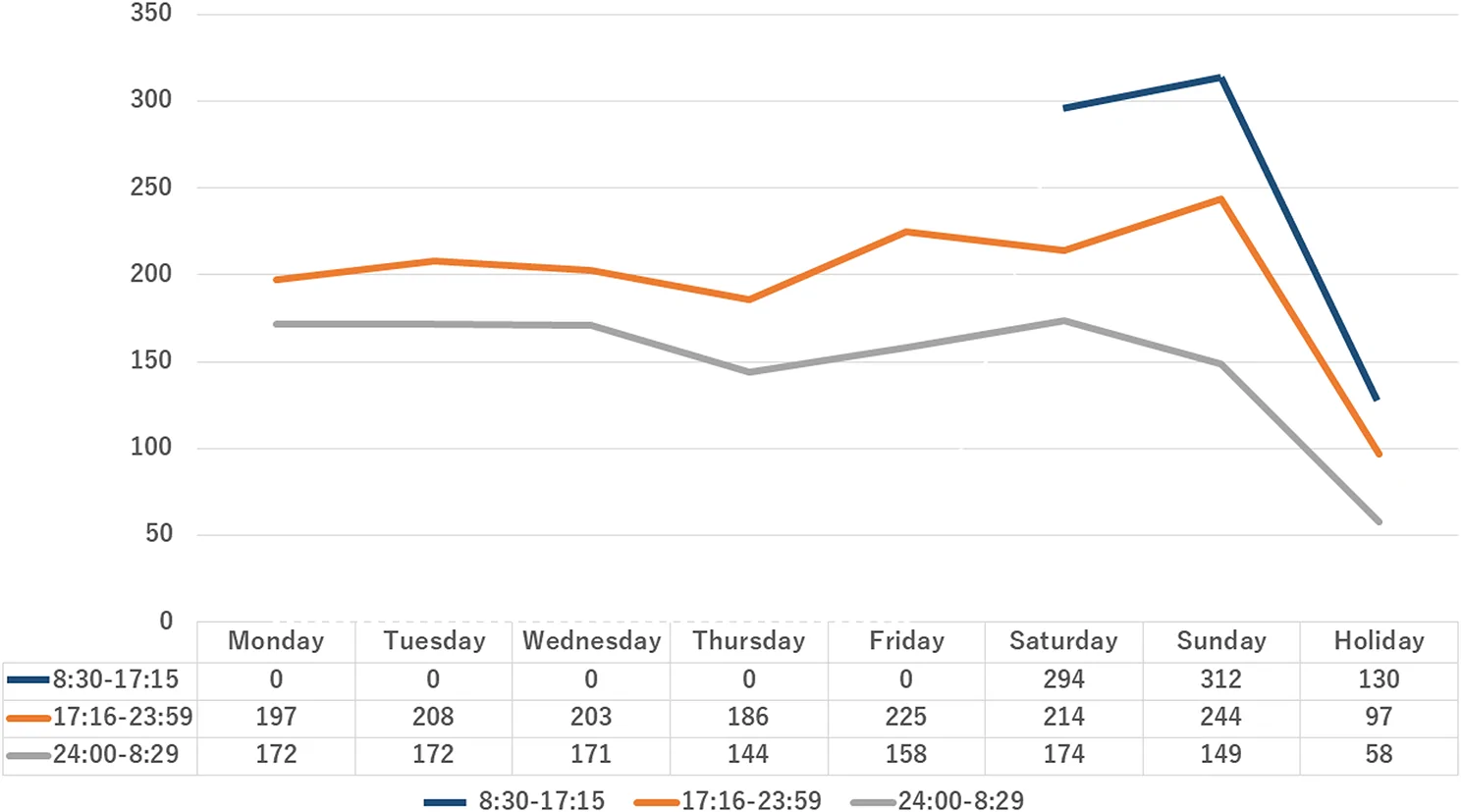
Temporal distribution of RRC calls across Off-hours. The figure illustrates the number of RRC requests received by SRCH during three time periods (08:30–17:15, 17:16–23:59, and 24:00–08:29) for each day of the week and national holidays. Weekday daytime hours (08:30–17:15) were excluded because RRC coverage during this period is shared among three tertiary hospitals, and the dataset included RRC dispatches only from the SRCH

Call volumes were concentrated during weekday nighttime hours, whereas weekends and national holidays showed consistently high call frequencies across both daytime and nighttime periods. Although weekday daytime data from other RRC-operating hospitals were incomplete, the available data were descriptively reviewed and showed cancellation rates comparable to those observed during off-hours. Overall, cancellation rates were similar across all time categories, remaining at approximately 30% regardless of weekday daytime, weekday nighttime, or weekend/holiday periods. The temporal distribution of RRC calls at SRCH is summarised in Fig. 4.

**Table 2:** Primary characteristics of the study participants for whom RRC was dispatched for pre-hospital emergency care (*n* = 3,508)

Variable	Cancelled Group (*n* = 1,091)	Treated Group (*n* = 2,417)	*p*-value
Age (mean of age ± SD)	59.1 ± 27.1	54.0 ± 27.7	< 0.001
Proportion treated (Children) *	128/482 (26.6%)	354/482 (73.4%)	0.020
Proportion treated (Adult)	963/3,026 (31.8%)	2,063/3,026 (68.2%)	0.020
Sex (male)	678 (62.2%)	1,538 (63.6%)	0.398
Requester			< 0.001
Command Centre (*n* = 2,620)	931 (35.5%)	1,689 (64.5%)	
Emergency Medical Service (*n* = 774)	159 (20.5%)	615 (79.5%)	
Judgement of the doctor (*n* = 114)	1 (0.9%)	113 (99.1%)	
Mean distance from hospital (km)	10.3 ± 6.7	7.5 ± 5.6	< 0.001
Distance to scene > 8.4 km	553 (50.7%)	753 (31.1%)	< 0.001
Median mission time (min)* *	23 [IQR: 14–35]	43 [IQR: 30–58]	< 0.001
Conditions			
Trauma (*n* = 653)	110 (16.8%)	543 (83.2%)	< 0.001
Cardiac arrest (*n* = 810)	238 (29.4%)	572 (70.6%)	0.229
Circulatory failure (*n* = 986)	373 (37.8%)	613 (62.2%)	< 0.001
Respiratory failure (*n* = 456)	208 (45.6%)	248 (54.4%)	< 0.001
Anaphylactic shock (*n* = 281)	129 (45.9%)	152 (54.1%)	< 0.001
Mass casualty incident (*n* = 21)	0 (0%)	21 (100%)	0.002
Others (*n* = 301)	33 (11.0%)	268 (89.0%)	< 0.001

*: Proportion treated = number of treated cases divided by the total number of requests within each age group

**: mission time, defined as the duration from dispatch to return arrival at SRCH (i.e., the working time for cancelled cases), was not normally distributed; therefore, it was reported using the median and IQR instead of the mean

### Age

The median age was 54.0 ± 27.7 years in the treated group and 59.1 ± 27.1 years in the cancelled group (*p* < 0.001). The cancellation rate was lower in paediatric patients (26.6%, 128/482) than in adults (31.8%, 963/3,026). Multivariable analysis showed a significant increase in cancellation rate among patients aged ≥ 65 years (OR, 1.39; 95% CI=1.22–1.66) (Tables 2 and 3).

### Diseases

The cancellation rates for respiratory failure and anaphylactic shock were relatively high, at 45.6% (208/456) and 45.9% (129/281), respectively. Among respiratory failure cases, 71.6% (149/208) of cancellations were classified as mild conditions.

Similarly, 76.0% (98/129) of anaphylactic shock cancellations were categorised as mild conditions (Tables 2 and 4).

### Distance

The average distance between the requested address and the hospital was 7.5 ± 5.6 km in the treated group and 10.3 ± 6.7 km (*p* < 0.001) in the cancelled group. The cancellation rate was significantly higher when the distance was farther than the average of 8.4 km. Among cancelled cases, those attributed to mild conditions had an average distance of 9.2 ± 6.3 km, whereas cases in which the RRC failed to reach the scene in time had an average distance of 12.2 ± 6.9 km (Tables 2 and 3, and 4).

### Types of requests

The lowest cancellation rate was observed in cases where physicians directly judged the need for dispatch, at 0.9% (1/114 cases). In contrast, on-site emergency teams had a cancellation rate of 20.5% (159/774 cases; OR 32.51; 95% CI, 4.44–233.07), whereas command centre-initiated requests had the highest cancellation rate at 35.5% (931/2,620 cases; OR 82.24; 95% CI, 11.41–592.70) (Tables 2 and 3).

### Factors independently associated with RRC cancellation

According to Tables 2 and 3, age ≥ 65 years (OR 1.39; 95% CI, 1.22–1.66), requests initiated by the command centre (931/2,620 cases; OR 82.24; 95% CI, 11.41–592.70), and greater distance from the hospital (OR 2.81; 95%CI, 2.40–3.28) were independently associated with cancellation.

**Table 3:** Multivariable logistic regression analysis of risk factors for cancellation of physician-staffed emergency medical services using rapid response cars

	Number of cases, *n*	Odds ratio*	95% CI	*p*-value
Age (years) **				
16–64	1,360	1		
≤ 15	482	0.95	0.75–1.20	0.64
≥ 65	1,660	1.39	1.22–1.66	< 0.01
Sex (male)	2,216	0.94	0.81–1.09	0.40
Requester				
Emergency Medical Service	774	32.51	4.44–233.07	< 0.01
Command Centre	2,620	82.24	11.41–592.70	< 0.01
Distance from the hospital (> 8.4 km)	1,306	2.81	2.40–3.28	< 0.01

*The reference group in Table 3 should be explicitly indicated as having an odds ratio of 1.0

**Age categories were defined as ≤ 15, 16–64, and ≥ 65 years. In Japan, pediatric departments commonly provide care up to 15 years of age, and individuals aged 65 years or older are officially classified as older adults. These cutoffs were adopted to reflect clinical practice and demographic classifications

**Table 4:** Details of reasons for cancellation of physician-staffed emergency medical services using rapid response cars

	Mild condition	Starting transport to another hospital	Others
Cancelled cases (n=1,091)	530 (48.6%)	461 (42.3%)	100 (9.2%)
Reason for request			
Trauma (*n* = 110)	61(55.0%)	37 (33.3%)	12 (10.8%)
Cardiac arrest (*n* = 238)	30 (12.6%)	146 (61.3%)	62 (26.1%)
Circulatory failure (*n* = 373)	180 (48.3%)	180 (48.3%)	13 (3.5%)
Respiratory failure (*n* = 208)	149 (71.6%)	52 (25.0%)	7 (3.4%)
Anaphylactic shock (*n* = 129)	98 (76.0%)	27 (20.9%)	4 (3.1%)

## Discussion

This study identified several key factors associated with higher RRC cancellation rates on off-hours. Univariate analyses revealed that increased cancellations were associated with advanced age, respiratory failure, anaphylactic shock, extended distances, and requests initiated by the command centre. Among these, multivariate logistic regression confirmed that age ≥ 65 years, requests initiated by the command centre, and greater distance from the hospital were independently associated with cancellation. To facilitate a structured interpretation of the findings, the following discussion is organised into five thematic sections: (1) patient and clinical characteristics, (2) request-related factors, (3) cancellation factors, (4) resource burden and cost implications, and (5) future considerations and research direction for RRC system optimisation. Each section explores the underlying mechanisms, contextual relevance, and potential implications for emergency medical practices.

### Patient and clinical characteristics

Cancellation rates were higher among individuals aged ≥ 65 years, which could be partly attributed to several clinical factors beyond their immediate physiological status. In addition to the absence of circulatory or respiratory failure at the time of assessment, the presence of Do Not Attempt Resuscitation (DNAR) orders or predefined advanced care plans likely influenced the decision-making process. Conversely, paediatric patients showed lower rates. This contrast may reflect differences in clinical urgency and decision-making dynamics: paediatric cases often prompt cautious dispatch due to rapid deterioration risks and caregiver anxiety, whereas older patients may present with non-specific symptoms or have care plans that evolve during transport coordination, leading to higher cancellation rates. In addition to these clinical considerations, operational factors also play a role. Paediatric emergencies often require transport to specialised facilities with limited availability, which may reduce the likelihood of cancellation once an RRC has been dispatched.

The five-year difference in mean age between the cancelled and treated groups is likely explained by the different proportions of paediatric patients in each group. Because children had a lower cancellation rate, the cancelled group contained fewer paediatric cases, which in turn increased the overall mean age.

Respiratory failure and anaphylactic shock had relatively high cancellation rates (45.6% and 45.9%, respectively) (Table 2). Although some reports have shown that RRC activities have contributed to a reduction in mortality from respiratory diseases [10, 17], these conditions often involve time-sensitive, yet potentially self-limiting episodes, such as choking and asthma, which may be promptly stabilised through pre-hospital or prescribed interventions. Several studies have highlighted the effectiveness of pre-hospital medicine in respiratory failure treatment [17, 18]. Similarly, many anaphylaxis-risk patients use autoinjectors (such as epinephrine) in pre-hospital situations, which are associated with a lower risk of hospitalisation and fatality [19]. Although some degree of over-triage is acceptable, these findings indicate that improved stratification of disease severity and pre-arrival responses may contribute to more efficient RRC deployment and a reduction in avoidable cancellations.

### Request-related factors

Evidently, the RRC request source significantly affects the cancellation rates. Requests made by the command centre showed the highest cancellation rate, whereas those made by on-site EMSs or physicians had lower rates. Notably, the ORs for command centre and EMS requests were extremely high (82.24 and 32.51, respectively). The elevated cancellation rate of command centre requests suggests that reviewing and refining dispatched keywords could lead to more efficient RRC operations. (Table 3).

### Cancellation factors

Cases in which the reason for cancellation was a ‘mild condition’ can be explained by the clinical stability of patients at the time of assessment. In the Japanese emergency medical system, such patients are typically transported to secondary care facilities, which are designated for moderate-severity cases with stable vital signs and no need for immediate physician-led interventions during transport. When the command centre dispatches an RRC based on specific “keywords” supplied by a caller, the patient’s actual clinical condition has not yet been confirmed. However, once on-site EMS personnel perform a direct assessment and determine that the patient is haemodynamically stable and is suitable for secondary care, specialised intervention by a physician-staffed RRC is often deemed unnecessary. In such cases, patients can be safely managed and transported by the EMS crew alone. Although rare, some cardiac arrest cases were classified as “mild” when resuscitation was deemed unnecessary on scene. However, because information on DNAR orders or other end-of-life directives was not available in our dataset, the specific reasons for these decisions could not be confirmed.

Additionally, cancellation rates increased in proportion to the distance between the emergency scene and the base hospital (Tables 2 and 3). One possible explanation is that longer response times enable confirmation of acceptance by a nearby facility before the RRC arrives. Nearby medical facilities are more likely to admit patients with time-sensitive conditions such as anaphylaxis or mild respiratory distress, which may improve with prescribed medication or early on-site treatment before the RRC arrives. In contrast, in trauma cases, previous reports have recommended that pre-hospital care ideally includes transportation to the most appropriate facility, regardless of the distance [12, 20]. This indicates the need to balance time constraints with clinical appropriateness when selecting destinations for RRC dispatch.

As shown in Fig. 3 and 70.9% of dispatch requests occurred during off-hours. Weekday daytime data from other RRC-operating hospitals were incomplete, but the available data were descriptively reviewed and showed cancellation rates comparable to those observed during off-hours. Although cancellation rates did not differ substantially across time periods or days of the week (29.9–31.6%), the concentration of calls during off-hours increases operational burden and may indirectly contribute to avoidable dispatches. Notably, Fig. 3 shows 225 patients with duplicated dispatches, indicating that these patients may have missed treatment opportunities because resources were allocated to cancelled cases.

### Resource burden and cost considerations

As shown in Table 2, each cancellation resulted in a median mission time of 23 min for at least three RRC crew members, without generating financial reimbursement. Although this duration appears short on an individual basis, each dispatch requires physicians and nurses to leave the emergency department, temporarily reducing in-hospital manpower. Therefore, even brief missions impose a significant operational burden.

Moreover, the cumulative impact is substantial. During the study period, 225 dispatch requests were not accepted because the RRC team was already engaged in another mission. Some of these unaccepted cases might have received timely physician-staffed pre-hospital care if unnecessary dispatches had been avoided. Because no RRC team was dispatched to these cases, patient-level clinical information was not available, and therefore, this group could not be described in further detail. This highlights that cancellation events should be evaluated not only by their duration but also by their contribution to opportunity loss within the emergency care system.

With fixed personnel costs, the cumulative opportunity loss and the impact on in-hospital patient care were significant. Although studies on helicopter EMS have assessed cost-effectiveness [21–24], data on the economic impact of RRC cancellations are limited. The American College of Surgeons recommends maintaining overtriage at < 35% and undertriage at 5% [25]. In this study, the cancellation rate was 31.1%, which is consistent with the findings of previous studies [1, 2, 10, 15, 16]. However, further efforts to minimise unnecessary dispatches are warranted to optimise emergency response efficiency.

### Future considerations and research direction for RRC system optimisation

Future improvements in RRC operations should focus on establishing evidence-based dispatch criteria, enhancing triage accuracy, and optimising real-time resource allocation. Regional variations in RRC systems—including differences in funding structures and operational models—highlight the need for coordinated approaches that reflect local demands while promoting system-wide consistency. Refining keyword-based dispatch criteria, particularly by identifying triggers with low predictive value or consistently high cancellation rates, may help reduce unnecessary activations and improve operational efficiency. In addition, clarifying the clinical added value of physician-staffed RRCs is essential for developing a sustainable and effective pre-hospital care model. Recent evidence demonstrating improved outcomes with physician-led pre-hospital teams underscores the importance of timely and appropriate deployment.

### Limitations

Before discussing these limitations, it is important to highlight the strengths of this study. To the best of our knowledge, this is the first investigation of physician-staffed RRC cancellations in an urban Japanese setting based on a large dataset of 5,652 dispatches across a population of nearly 2 million. These findings offer valuable insights into operational inefficiencies and provide a foundation for future system improvements. Overall, both the internal and external validity of this study are limited due to its retrospective single-centre design.

#### Internal validity

Some limitations of this study may have influenced its internal validity. Missing data, particularly among cancellation cases, and the exclusion of duplicate requests may have introduced selection bias. Additionally, the use of straight-line distance as a proxy for response time did not account for real-world factors such as road conditions, traffic, or vertical location within buildings. These factors may have affected the precision of the time-related evaluations. Furthermore, the study did not evaluate the dispatch keywords used in triage decisions, limiting insight into the appropriateness of case selection. Information on DNAR orders was also unavailable in our dataset, preventing us from evaluating their prevalence or potential influence on cancellation decisions.

#### External validity

The findings are based on a single urban region where SRCH serves as the sole trauma centre, which may limit generalisability to areas with different emergency transport policies or multiple receiving facilities. The COVID-19 pandemic may have further influenced dispatch patterns, particularly for respiratory cases. Moreover, because the national RRC registry was launched only recently (February 2024), comparative data remain limited. Regional disparities in pre-hospital infrastructure also complicate broader extrapolation.

### Future directions

Despite these limitations, this study provides a valuable foundation for improving RRC operations. Detailed information was not available for the 225 cases in which an RRC dispatch request was made but the unit did not depart. Therefore, the reasons for non-dispatch and the potential for under-triage could not be evaluated. These data are highly relevant for policy makers, and future research should incorporate information on denied-dispatch cases to enable a more comprehensive assessment of dispatch appropriateness. Future research should incorporate real-time dispatch data, evaluate keyword-based triage criteria, and include multi-regional comparisons to validate and extend these findings.

## Conclusions

Several factors were associated with higher cancellation rates of physician-staffed RRC dispatches during off-hours, including advanced age, respiratory failure, anaphylactic shock, longer distances, and requests originating from command centres. These findings underscore the need to improve dispatch accuracy and reduce avoidable cancellations. Future research should prioritise the development of evidence-based dispatch criteria and further clarify the added value of physician-staffed RRCs in pre-hospital care systems. Establishing evidence-based dispatch criteria is essential for building a sustainable and effective physician-staffed pre-hospital care system.

## Data Availability

The data supporting the findings of this study are available upon reasonable request.

## Abbreviations

CI: Confidence interval
DNAR: Do not attempt resuscitation
IQR: Interquartile range
OR: Odds ratio
P-EMSs: Physician-staffed emergency medical services
RRC: Rapid response car
SRCH: Saitama Red Cross Hospital
SD: Standard deviation
